# Comparative Bioactivity Analysis of Green-Synthesized Metal (Cobalt, Copper, and Selenium) Nanoparticles

**DOI:** 10.7759/cureus.55933

**Published:** 2024-03-11

**Authors:** Iadalin Ryntathiang, Mukesh Kumar Dharmalingam Jothinathan, Archana Behera, Saantosh Saravanan, Ramadurai Murugan

**Affiliations:** 1 Centre for Global Health Research, Saveetha Medical College and Hospitals, Saveetha Institute of Medical and Technical Sciences, Chennai, IND

**Keywords:** selenium nanoparticles, copper nanoparticles, cobalt nanoparticles, green synthesis, antibacterial activity, nanotechnology

## Abstract

Aim

This study involves synthesizing metal nanoparticles (NPs) via the green synthesis method using *Millettia pinnata* leaf, *Acacia auriculiformis* bark, and *Citrus sinensis* peel and comparatively evaluating their antibacterial activity in vitro through the analysis of cobalt oxide NPs (CoNPs), copper NPs (CuNPs), and selenium NPs (SeNPs). This research contributes to eco-friendly approaches for producing functional nanomaterials with potential applications in medicine and environmental remediation.

Materials and methods

The metal NPs were synthesized using *M. pinnata* leaf, *A. auriculiformis* bark, and *C. sinensis* peel. These leaf extracts act as self-reducing and stabilizing agents. The antibacterial activity was assessed by the well diffusion method. Cultures of pathogenic bacteria species such as *Staphylococcus aureus*, *Escherichia coli*, *Bacillus subtilis*, and *Pseudomonas aeruginosa* were prepared. NPs were applied to the culture, and zones of inhibition (ZOIs) were measured. The data were statistically analyzed to compare the antibacterial efficacy of the different NPs.

Results

The successfully synthesized CoNPs, CuNPs, and SeNPs showed distinctive phytochemical properties. CoNPs exhibited the highest ZOI against most bacterial strains, with CuNPs and SeNPs following. CoNPs consistently showed superior performance compared to CuNPs and SeNPs.

Conclusion

Our study analyzed the bioactivity of metal NPs produced using green synthesis with plant extracts. CoNPs have shown superior antibacterial effectiveness against both Gram-positive and Gram-negative bacteria when compared to CuNPs and SeNPs. This may be due to their larger surface area, smaller size, unique electrical, magnetic, and catalytic properties, as well as their improved contact with the bacterial cell wall and membrane.

## Introduction

Nanotechnology has evolved quickly and finds many applications in a wide range of areas, including bioengineering, chemistry, biology, and physics. Nanoparticles (NPs) are interesting because of their distinct characteristics in size and shape, which are produced through top-down and bottom-up approaches [1,2]. Bottom-up approaches involve several ways, with physical techniques requiring elevated temperature, pressure, and costs. Most chemical procedures utilize dangerous substances that are harmful to biological systems and their surroundings. The biological method is an eco-friendly and biocompatible method increasingly used for producing NPs [3].

Over the past decade, modern nanotechnology processes have drawn more attention due to the low cost of inorganic metals as precursors for antibacterial agents. These metals are highly selective, exhibit resistance to high temperatures, and have long-lasting stabilized elements compared to other organic agents [4]. Various applications utilize NPs like zinc oxide, gold, selenium, silver, sulfide, platinum, cerium oxide, titanium oxide, chitosan, palladium, cellulose, silica, iron, antimony trioxide, zirconium dioxide, and cobalt. These NPs exhibit potential in biomedical applications because of their antibacterial and antioxidant characteristics and their capability to transition between oxidation states [5,6]. Previous studies have demonstrated the significant potential of plant extract-based metal NPs for treating bacterial, fungal, and dermatological diseases [3].

Selenium NPs (SeNPs) are also known as crucial trace elements for maintaining the health of humans and animals. Kondaparthi et al. reported that selenium exhibits antimicrobial, antidiabetic, antioxidant, and anti-inflammatory properties [7]. Similarly, cobalt NPs (CoNPs) are environmentally friendly and have a wide range of applications, including pharmaceuticals, cosmetics, environmental remediation, pigments, and dyes [4]. Due to their unique characteristics, nano-copper has been the focus of research efforts over the past decade. Copper NPs (CuNPs) offer advantages such as nontoxicity, high contact surface area, stability, and a wide range of possible applications as antibacterial agent, detector, catalyst, and optical sensor [8,9].

In NPs synthesizing, the biological approach utilizes microorganisms or medicinal plants, especially those with therapeutic properties, which is more cost-effective and eco-friendly compared to other approaches. During the extraction process, plant phytocompounds, well known for their antioxidant qualities, can be included in NPs [10]. More than 200 species of plants in the genus Millettia, which belongs to the Fabaceae family, are cultivated worldwide in tropical and subtropical climates [11]. *Millettia* *species* have been traditionally used for their antitumor, antibacterial, pesticidal, insecticidal, chemopreventive, and antispasmodic properties in rheumatoid arthritis, treating joint pain, tuberculosis, and amenorrhea, among other diseases [12,13].

*Acacia auriculiformis*, belonging to the Fabaceae family, is a medium-sized, straight, deciduous, or evergreen tree that may grow up to 30 m in height. It is typically found in parks and roadside areas in India. The common name “acacia” is derived from the Greek word “akis,” meaning a spike or point. Plants have shown a diverse range of pharmacological activities such as hepatoprotective, wound-healing, antibacterial, antimalarial, antifilarial, cestocidal, antimutagenic, chemopreventive, and spermicidal properties [14].

Citrus juice extraction results in a large percentage of goods and essential residues, such as peel and segment membranes. These residues, abundant in proteins, bioflavonoids, polyphenolics, and other chemicals, have been found useful as stabilizing and reducing agents for synthesizing nanomaterials. Specifically, chemicals known as polyphenolics are gaining prominence due to their substantial biological activity, including antibacterial, antioxidant, and anti-inflammatory qualities [15].

This research aims to evaluate and compare the antibacterial activity of CoNPs using *Millettia pinnata*, CuNPs using *A. auriculiformis*, and SeNPs mediated by *Citrus sinensis*. By combining green synthesis with the antibacterial qualities found in medicinal plants, this study aims to identify combinations that can be utilized in potential applications in health and related industries. The study of the complex interactions between nanotechnology and natural sources is enhanced by evaluating and comparing the antibacterial activity of CoNPs, CuNPs, and SeNPs mediated by specific medicinal plants. These findings might result in the development of innovative antibacterial alternatives, with wider implications for environmentally friendly and sustainable biomedical applications.

## Materials and methods

The study was conducted at the Centre for Global Health Research, Saveetha Medical College and Hospitals, Saveetha Institute of Medical and Technical Sciences, Chennai, India.

Collection of materials

Fresh leaves of *M. pinnata *were collected from Chennai. The bark of* A. auriculiformis *was collected from Balasore, Orissa, and *C. sinensis* (orange) fruits were purchased from a fruit shop in Tambaram, Chennai. The plant* M. pinnata *leaves and *A. auriculiformis* bark samples were authenticated by the Centre for Advanced Studies in Botany at the University of Madras, Chennai, India.

Preparation of samples

To remove all the fine dust, the *M. pinnata* leaves,* A. auriculiformis* bark, and *C. sinensis* peels were thoroughly washed with running water three times and then rinsed with distilled water to eliminate all fine dust. The samples were then diced into small fragments and left to shade-dry for three days. Afterward, they were mechanically ground into a powder using a blender. The powdered samples were stored for further experiments [16].

Preparation of aqueous extracts

About 10 g of dried* M. pinnata* leaf powder was mixed with 100 mL of distilled water in a conical flask. The mixture was heated to 60°C for 20 minutes using a heating mantle while being continuously stirred and later kept in a stirrer. The liquid was cooled and filtered through muslin fabric after being heated to remove debris, and the filtrate was then collected. The filtrate was further purified by filtering it through the Whatman No. 1 filter paper to eliminate any remaining particles. The aqueous extract was stored at -40°C in a refrigerator for future analysis. The same procedure was followed for the preparation of *A. auriculiformis* bark and *C. sinensis* peel, respectively.

Preparation of metal solutions

A 1 Mm cobalt chloride (CoCl_2_) stock solution was prepared by dissolving it in 100 mL of distilled water. A 3 Mm copper chloride (CuCl_2_) stock solution was prepared by dissolving it in 100 mL of distilled water. A 3 Mm sodium selenite (Na_2_SeO_3_) stock solution was prepared by dissolving it in 100 mL of distilled water.

Instruments

The instruments used in this study include Labman Scientific UV-visible spectrophotometer (200-400 nm, Chennai, India), JSM-IT800 NANO SEM, Thermo Fisher Scientific Nicolet Summit LITE Fourier transform infrared spectroscopy (FTIR) Spectrometer (400-4000 cm^-1^, Waltham, Massachusetts, United States), a hot air oven (Techno/EBI, Techno Instruments Company, Bengaluru, India), a global digital pH meter, an autoclave (Labman/LMUC, Labman Scientific Instruments Pvt. Ltd., Chennai, India), an incubator (New Lab Equipments, Coimbatore, India), a laminar airflow chamber (New Lab Equipments), a shaking incubator (New Lab Equipments), a water bath (Techno/EBI, Techno Instruments Company), and a REMI 1MLH stirrer (REMI Lab World, Mumbai, India).

Chemicals

The chemicals used in this study include CoCl_2_ (1 Mm) (Sisco Research Laboratories Pvt. Ltd., Mumbai, India), CuCl_2_ (3 Mm) (Sisco Research Laboratories Pvt. Ltd.), Na_2_SeO_3_ (3 Mm) (Sisco Research Laboratories Pvt. Ltd.), ethanol (Sisco Research Laboratories Pvt. Ltd.), and Mueller-Hinton agar (HiMedia Laboratories Private Limited, Thane, India).

Biosynthesis preparation of metal NPs

**Figure 1:**
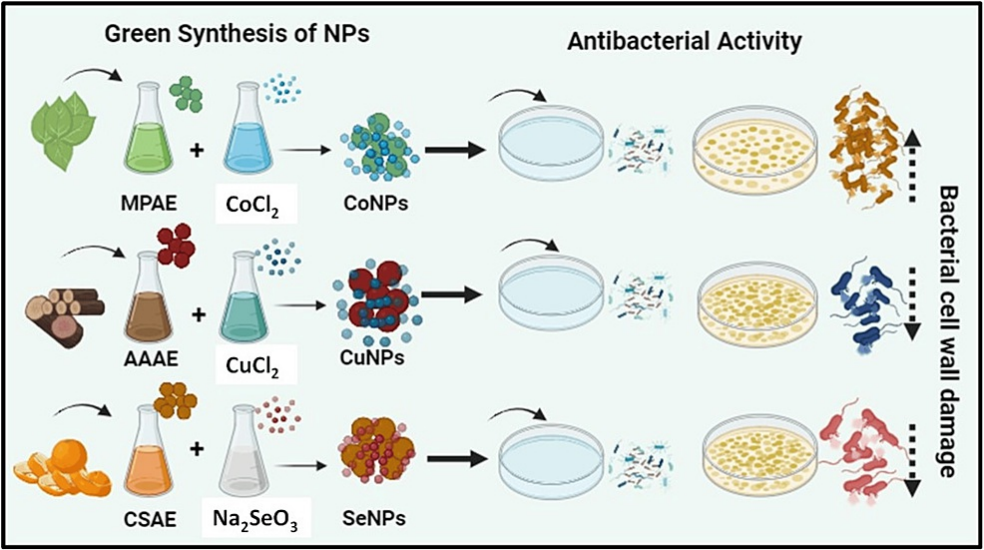
Schematic representation of different metal nanoparticles preparations and their responses to antibacterial activity *Acacia auriculiformis* aqueous extract (AAAE), *Citrus sinensis* aqueous extract (CSAE), ​​​​​​​*Millettia pinnata* aqueous extract (MPAE), selenium nanoparticles (SeNPs), cobalt nanoparticles (CoNPs), copper nanoparticles (CuNPs)

For the synthesis process, CoNPs, CuNPs, and SeNPs were prepared separately. Each metal precursor solution was mixed with distilled water and stirred. Following this, *M. pinnata* aqueous extract (MPAE), *A. auriculiformis *aqueous extract (AAAE), and* C. sinensis* aqueous extract (CSAE) were added individually, resulting in observable color changes. After that, the mixtures were placed in an incubator at 37°C for a duration of four days. The color change observed after this incubation period indicated successful NPs synthesis. Control without metal precursor solutions showed no color change. Figure 1 illustrates a visual observation of metal NPs preparations and their responses to antibacterial activity.

Metal NPs characterization

By using a Labman Scientific Ultraviolet-visible spectrophotometer, the UV-vis spectra of the produced NPs were observed at different reaction times. The instrument operated in the 200-400 nm range with a specified resolution. A FTIR spectrometer was used to perform FTIR over the 400-4000 cm^-1^ range. This facilitated the identification and assignment of various vibration modes, enabling the determination of distinct functional groups in MPAE, AAAE, and CSAE.

Antibacterial activity

Collection of Test Bacterial Species

Collected cultures include two Gram-positive bacteria (*Bacillus subtilis *and *Staphylococcus aureus*) and two Gram-negative bacteria (*Escherichia coli* and *Pseudomonas aeruginosa*).

The antibacterial activity test was performed using the method described by Das and Saikia. The Mueller-Hinton agar was prepared, sterilized at 15 lbs of pressure for 20 minutes, and then cooled to 45°C. The medium was poured onto sterile petri plates and left to solidify. The plates, which contained the media, were then inoculated with the required microbial culture using sterile swabs. Sterile well cutters were used to cut wells in the agar [17]. Subsequently, 50 µL of CoNPs, CuNPs, and SeNPs, aqueous extracts (MPAE, AAAE, and CSAE), and a control (gentamicin) were added to each well. The plates were kept at 37°C for 24 hours. After incubation, the diameter of the zone of inhibitions (ZOIs)was measured and recorded in millimeters (mm).

## Results

**Figure 2:**
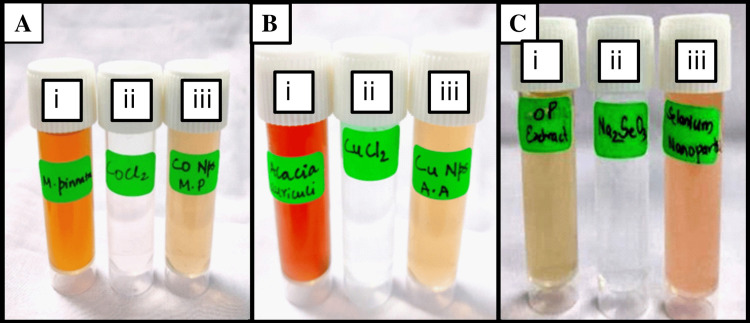
Photocopy images of reactants to product formation in metal NPs: (A) Cobalt nanoparticles, (B) Copper nanoparticles, and (C) Selenium nanoparticles

The successful synthesis of CoNPs by a biological method was observed, wherein we noted color changes confirming the formation of NPs as shown in Figure 2A. Initially, the MPAE extract exhibited a light orange color (Figure 2A(i)), while the metal precursors appeared colorless (Figure 2A(ii)). After the reaction was complete, the solution turned light brown, as depicted in Figure 2A(iii). No further color changes occurred following four hours of incubation at 25°C. Throughout the synthesis, MPAE played a dual role as both a reducing and a stabilizing agent. Upon introducing MPAE to a clear CoCl_2_ solution, the solution rapidly turned light brown, indicating the successful formation of CoNPs after 45 minutes, as depicted in Figure 2A(iii).

The biological synthesis of CuNPs was effectively completed, and the color changes observed signify the NPs formation as depicted in Figure 2B. The AAAE extract initially appears as dark brown in Figure 2B(i), whereas the metal precursors are visible as colorless in Figure 2B(ii). Once the reaction was completed, the solution turned light brown, as shown in Figure 2B(iii). CuNPs were produced simply and sustainably through this technique. Throughout the synthesis, AAAE plays the important role of a stabilizing and reducing agent. After 45 minutes, the clear CuCl_2 _solution quickly turned light brown upon the addition of AAAE, indicating the effective production of CuNPs, as shown in Figure 2B(iii).

The successful synthesis of SeNPs by a biological method is evidenced by the observed color changes, confirming the formation of NPs as shown in Figure 2C. Initially, the CSAE extract exhibits a light grey color (Figure 2C(i)), while the metal precursors appear colorless (Figure 2C(ii)). After the completion of the reaction, the solution turns light brown, as depicted in Figure 2C(iii). CSAE plays a dual role as both a reducing and stabilizing agent. When CSAE is introduced to a clear Na_2_SeO_3 _solution, the solution rapidly turns light brown, indicating the successful formation of SeNPs after 45 minutes, as shown in Figure 2C(iii).

Characterization of CoNPs, CuNPs, and SeNPs

Ultraviolet-Visible Spectroscopy

**Figure 3:**
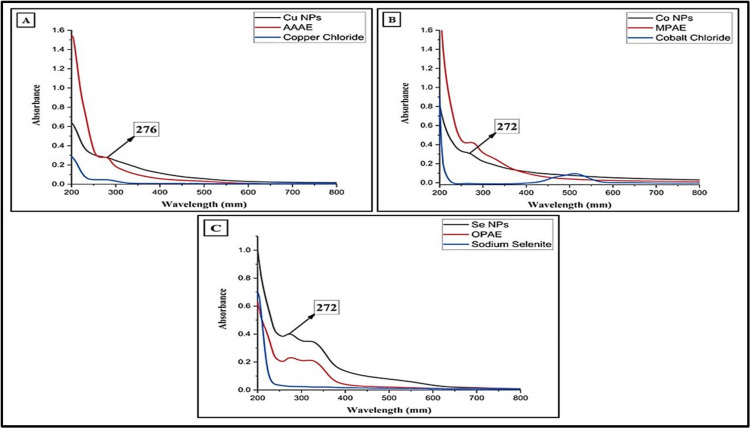
Ultraviolet-visible spectrum of (A) Copper nanoparticles synthesized using AAAE, (B)Cobalt nanoparticles synthesized using MPAE, and (C) Selenium nanoparticles synthesized using CSAE *Millettia pinnata* aqueous extract (MPAE), *Acacia auriculiformis* aqueous extract (AAAE), ​​​​​​​*Citrus sinensis* aqueous extract (CSAE)

In this study, plant extracts with medicinal importance were subjected to phenotypic screening. This study showed that the bark of *A. auriculiformis* has this capability. The formation of CuNPs was confirmed by analyzing their UV-vis spectra. The highest absorption peak observed at ~276 nm confirms that AAAE interacted with the metal to form CuNPs after overnight development. In this spectrum, the blue line shows the pure copper metal precursor, the red line represents AAAE, and the black line confirms the presence of CuNPs. The peak at around ~276 nm corresponds to the sp3 hybridization of CuNPs, as depicted in Figure 3A.

The findings of this research revealed that the leaf extract of *M. pinnata *possesses this ability. Through analysis of their UV-vis spectra, as shown in Figure 3B, the blue color line in this spectrum reveals the presence of a pure cobalt metal precursor. The absorption peak around 400-500 nm clearly confirms the d-d transition of the metal precursor. The red line indicates the presence of MPAE, while the black line signifies the presence of CoNPs. The confirmation of the reaction of CoNPs was verified by the shift in wavelength to around ~272 nm.

The study’s findings demonstrated that *C. sinensis* leaf extract displayed this property. We confirmed SeNPs’ reaction by examining their UV-vis spectra, as illustrated in Figure 3C. The presence of a pure selenium metal precursor is indicated by the blue line, the CSAE is represented by the red line, and the presence of SeNPs is confirmed by the black line. After being incubated overnight, a maximum absorption peak at around ~272 nm emerged, indicating the interaction between the metal and the CSAE, resulting in the formation of SeNPs. The peak at ~272 nm indicates the presence of sp3 hybridization of SeNPs.

FTIR

**Figure 4:**
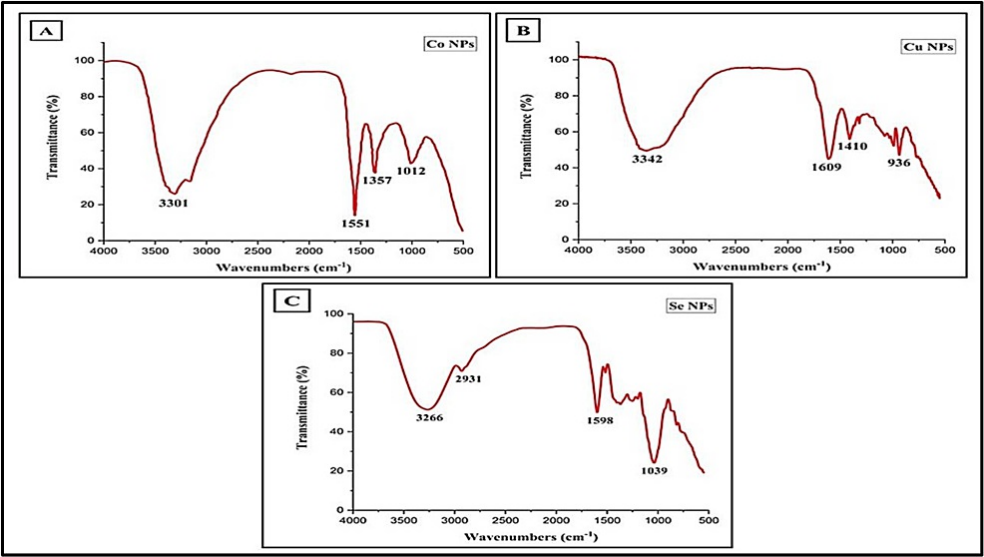
FTIR spectrum of (A) Cobalt nanoparticles synthesized using MPAE, (B) Copper nanoparticles synthesized using AAAE, and (C) Selenium nanoparticles synthesized using CSAE *Millettia pinnata* aqueous extract (MPAE), *Acacia auriculiformis* aqueous extract (AAAE), ​​​​​​​*Citrus sinensis *aqueous extract (CSAE)

FTIR spectroscopy is a common technique used to determine functional groups based on their specific wavenumbers, ranging between 400 and 4000 cm^-1^. Within natural product research, this spectroscopic method is sufficient for identifying bioactive compounds. The spectra displayed a variety of peaks at various wavenumbers in CoNPs. Figure 4A showed a prominent peak at 3301 cm^-1^. The significant peak at 3301 cm^-1 ^represents N-H stretching, and the distinct peak at 1551 cm^-1^ corresponds to N-O stretching. The peak at 1357 cm^-1 ^signifies C-H bending, while the peak at 1012 cm^-1^ indicates C-F stretching. Phytochemicals such as flavonoids, alkaloids, steroids, terpenoids, and phenols were found in abundance in the *M. pinnata* extract.

The FTIR spectrum provides details about the functional groups in the produced CuNPs utilizing *A. auriculiformis*. FTIR spectra showed distinct peaks at various wavenumbers, with a significant peak at 3342 cm^-1^ in Figure 4B. The prominent signal at 3342 cm^-1^ represents O-H stretching vibrations, suggesting the existence of absorbed water molecules from the plant extract. A notable peak at 1609 cm^-1^ represents C=C stretching (α, β-unsaturated ketone), while peaks at 1410 cm^-1^ and 936 cm^-1^ suggest S=O stretching and C=C bending, respectively. CuNPs demonstrate interactions with phenolic compounds, alkenes, amines, terpenoids, and flavonoids.

Similarly, FTIR analysis was conducted to synthesize SeNPs with a *C. sinensis *extract as a capping agent. The analysis identified stable synthesized SeNPs, which exhibited significant peaks at 3266 cm^-1^ as illustrated in Figure 4C. The peak at 3266 cm^-1^ represents O-H stretching vibrations, and the peak at 2931 cm^-1^ indicates C-H stretching. The peak at 1598 cm^-1^ signifies N-H bending, while the peak at 1039 cm^-1^ signifies C-O stretching.

Antibacterial activity of CoNPs, CuNPs, and SeNPs

The antibacterial analysis presented here demonstrates the efficacy of metal NPs synthesized from various plant extracts, as shown in Table 1. CoNPs derived from MPAE had notable antibacterial effects against *S. aureus*, *P. aeruginosa*, and *E. coli*, with the largest zone of inhibition (ZOI) measuring 17 mm. *B. subtilis *showed a ZOI of 15 mm. CuNPs synthesized from AAAE exhibited the highest ZOIs against* S. aureus* (13 mm) and comparable activity against other pathogens. The SeNPs derived from CSAE displayed notable antibacterial activity against *B. subtilis*, with a maximum ZOI of 19 mm, and *E. coli*, with ZOIs of 16 mm, followed by *S. aureus* and *P. aeruginosa*, both showing ZOIs of 15 mm. In contrast, controls with plant extracts showed no ZOIs. These results suggest that NPs synthesized from plant extracts possess antibacterial properties comparable to conventional antibiotics like gentamicin.

**Table 1:** ZOI (mm) of metal NPs against bacterial strains tested

S. no	Bacteria name	Zone of Inhibition (mm)								
		*Millettia pinnata *extract	CoNPs	Control	*Acacia auriculiformis* extract	CuNPs	Control	*Citrus sinensis *extract	SeNPs	Control
1	Staphylococcus aureus	-	17 mm	19 mm	-	13 mm	24 mm	-	15 mm	15 mm
2	Escherichia coli	-	17 mm	19 mm	-	10 mm	19 mm	-	16 mm	12 mm
3	Bacillus subtilis	-	15 mm	17 mm	-	12 mm	18 mm	-	19 mm	13 mm
4	Pseudomonas aeruginosa	-	17 mm	15 mm	-	11 mm	21 mm	-	15 mm	11 mm

## Discussion

This paper aims to compare the antibacterial activity of CoNPs, CuNPs, and SeNPs synthesized from *M. pinnata* leaf, *A. auriculiformis* bark, and *C. sinensis* peel, respectively. The objective is to determine their potential as novel antibacterial agents. The study aims to determine the impact of different plant materials on the characteristics of NPs and their interactions with bacterial cells to improve antibacterial treatments. The research seeks to make substantial advances in the field of green nanotechnology and its applications in medical science.

CoNPs were produced using plant nutrients. FTIR analysis showed peaks at 3261 cm^-1^ and 1636 cm^-1^, indicating the presence of O-H stretching and amides. Plant-derived compounds are essential for capping, stabilizing, and reducing NPs, as indicated by peak at 1382 cm^-1^ and 1639 cm^-1^. Additionally, the presence of carbon and OH molecules enhances the reduction process and the effectiveness of CoNPs, emphasizing the importance of plant-based synthesis techniques in producing nanomaterials [18].

Similarly, in the case of metal NPs synthesis often involves a complex interplay between the biomolecules present in the metal ions and the plant extract, leading to distinctive optical and spectroscopic characteristics. In CuNPs production, the color change is caused by the interaction between conduction electrons in the NPs and incoming photons, as indicated by the presence of a distinct peak at around ~269 nm in the UV-vis spectrum. The FTIR spectra of CuNPs show bands at 3264 cm^-1 ^and 1636 cm^-1^, corresponding to those in the leaf extract, suggesting the existence of flavonoids and other phenolic substances. The biomolecules probably transition from an enol form to a keto form, aiding in the reduction of Cu²⁺ ions and the synthesis of CuNPs. The stability of CuNPs is likely due to the chelation between flavonoids and metal ions, as demonstrated in previous work by Mali et al. [9].

In contrast, the synthesis of SeNPs presents distinct spectroscopic features. The UV-vis spectrum of SeNPs displays a significant absorption peak at ~277 nm, indicating their characteristic optical properties. The FTIR spectrum further elucidates the swift synthesis of SeNPs, with vibration peaks corresponding to various functional groups, such as O-H stretch, C-H stretch, nitro compounds, aromatics, alkanes, and other reducing and stabilizing agents. These vibrational peaks provide insights into the mechanisms involved in SeNPs synthesis and the diverse biomolecules participating in the process [19].

Because of their nonpolar characteristics, numerous antimicrobial substances found in plant extracts are unable to diffuse across the aqueous agar matrix used in agar diffusion experiments. Furthermore, variables such as the extract’s concentration, the extraction process, and the presence of additional chemicals that could disrupt the effectiveness of bioactive substances can also impact the final result. It is important to take into account the particular microorganism being examined, as certain ones may have natural resistance to the components found in the plant extract [20].

CoNPs possess strong antibacterial characteristics due to their capacity to produce reactive oxygen species, disrupt microbial membranes, and interfere with crucial cellular processes. The size, shape, and surface charge of CoNPs can enhance their interaction with bacteria, resulting in increased antibacterial activity [21]. SeNPs also exhibit antibacterial capabilities; however, they may not be as efficient as CoNPs in producing ZOIs [22]. This variation could be attributed to changes in the mechanism of action, with SeNPs potentially exhibiting less reactivity or a decreased affinity for binding to bacterial cells. CuNPs are known for their antibacterial properties due to the production of copper ions, which induce oxidative stress and harm bacterial cells. The antimicrobial efficacy of CuNPs can be affected by variables such as particle size, concentration, and the presence of other chemicals. Recent studies have shown that the antibacterial effectiveness of CoNPs is notably superior to that of SeNPs and CuNPs. This increased impact of CoNPs is likely due to their reduced size, which enables improved penetration and interaction with bacterial cells, leading to more prominent inhibitory zones. Additionally, the distinctive electrical structure and oxidative characteristics of cobalt may enhance its exceptional antibacterial effects [23].

## Conclusions

Our work compared the bioactivity of metal NPs synthesized through green synthesis using plant extracts. Among the three types of NPs tested (cobalt, copper, and selenium), CoNPs showed the highest antibacterial efficacy against both Gram-positive and Gram-negative bacteria compared to copper and SeNPs. This could be attributed to their greater surface area, smaller size, distinctive electrical, magnetic, and catalytic characteristics, as well as their enhanced interaction with the bacterial cell wall and membrane. In comparison, selenium and CuNPs may exhibit different levels of activity due to variations in their inherent characteristics, such as electrical configuration, surface reactivity, and catalytic behavior. Moreover, CoNPs exhibited strong anti-inflammatory and antioxidant properties, which could be advantageous for various biomedical applications. As a result, the green production of CoNPs using plant extracts is a feasible and environmentally friendly method for producing effective and biocompatible nanomaterials.
